# Efficacy of *Garcinia kola* 0.5% Aqueous Eye Drops in Patients with Primary Open-Angle Glaucoma or Ocular Hypertension

**DOI:** 10.4103/0974-9233.61224

**Published:** 2010

**Authors:** Adebukunola O. Adefule-Ositelu, Bernice O. Adegbehingbe, Adebayo K. Adefule, Olayinka O. Adegbehingbe, Elsie Samaila, Kehinde Oladigbolu

**Affiliations:** Department of Ophthalmology, Lagos University Teaching Hospital, Idi-Araba, Lagos; 1 Departments of Surgery, (Ophthalmology Unit) and Orthopedic Surgery and Traumatology, Obafemi Awolowo University, Ile-Ife, Nigeria; 2 National Eye Centre, Kaduna

**Keywords:** *Garcinia kola*, *Glaucoma*, Iop Lowering Effect, Timolol

## Abstract

**Purpose:**

To evaluate the intraocular pressure (IOP) lowering efficacy of *Garcinia kola* 0.5% aqueous solution eye drops in patients with newly diagnosed primary open-angle glaucoma or ocular hypertension (POAG/OH).

**Materials and Methods:**

A randomized, double-masked, multicenter, active-controlled prospective study. Patients who met the inclusion criteria were randomly assigned in equal numbers to receive Timolol 0.5% eye drops as a control medication (A = Group 1 eyes) or *Garcinia kola* 0.5% eye drops as the study medication (B = Group 2 eyes). All drops were instilled at 6 am and 6 pm daily. Goldman applanation tonometry was performed at 9 am, 12 pm and 3 pm at baseline, week-6, week-12 and week-24 visits. Voluntary and actively elicited reports of adverse events were documented. The mean change in IOP over 24 weeks was the primary outcome measure. Both groups were compared for statistically significant differences at all visits. A *P* < 0.05 was considered statistically significant.

**Results:**

A total of 178 patients were randomly assigned to *G. kola* and Timolol groups. At baseline there were no differences in mean IOP between groups, based on age, sex, or diagnosis. At the end of the study period (24^th^ week), the mean (± SD) reduction in IOP was 12.93 ± 2.3 mmHg (47.8% ± 0.8% reduction) in *G*. *Kola* group and 13.09 ± 2.8 mm Hg (48.2% ± 1.03% reduction) in the Timolol group (*P* > 0.05). Adverse events were mild in nature with no statistically significant differences between groups (*P* > 0.05).

**Conclusions:**

*Garcinia kola* ophthalmic solution significantly reduces IOP as compared to baseline. The IOP lowering effect of both treatments was equivalent.

## INTRODUCTION

Glaucoma is a leading cause of irreversible blindness. The progression of this disease can be reduced or stopped by lowering IOP with medication and surgery.^1^ Among the several options available for medical therapy of elevated IOP, topical β-blockers and the prostaglandin F2α analogs, such as latanoprost, are the most commonly prescribed first-line therapies.^2^,^3^

In Nigeria, the most commonly available IOP reducing agents are β-blockers (mainly timolol maleate 0.5% eye drops) and prostaglandins (mainly latanoprost 0.005%). Latanoprost 0.005% is more effective than timolol maleate 0.5% in reducing diurnal IOP fluctuations. This is clinically important because optic nerve damage is accelerated by IOP fluctuations.^4^,^5^ However, both drugs are expensive and there is poor compliance with long-term use in Nigerian patients. Several studies have compared the efficacy of other topical glaucoma treatments in various settings.^6^–^10^ Our current challenge was to search for an alternative medication that is affordable, available and effective in lowering IOP in a limited resource economy.

*Garcinia kola* Heckel of the family Guttiferaceae is an indigenous herb in Nigeria colloquially referred to as “bitter kola”, “false kola” or “male kola.” *Garcinia kola (G. kola)* has anti-inflammatory, anti-parasitic, antimicrobial and antiviral properties.^11^,^12^ A recent study reported a reduction in subchondral pressure and pain in knee osteoarthritis due to *G. kola*.^12^ *G. kola* has also proven safe in human eyes^13^–^15^ and its systemic blood pressure lowering effects has been published in previous studies.^16^,^17^ During the initial stages of this study, Alcon Research Laboratories Inc., USA, was consulted to perform chromatography and pharmacodynamics analyses of the *G. kola* nut extract. Results of analyses indicated that *G. kola* extract inhibits the active process of aqueous secretion mediated through selective ion transport across basolateral membranes of non-pigmented ciliary epithelium. The enzymes involved in the transport are sodium potassium activated adenosine triphosphate (Na^+^ K^+^ ATPase) and carbonic anhydrase.^18^ The former is found predominantly in plasma membranes of basolateral infoldings of the NPE. Some have proposed the IOP lowering activity of *G. kola* is a combination of a vasodilation effect, which reduces aqueous production through the lowering of perfusion pressure, and a miotic effect which increases outflow facility.^19^

The aim of this study was to compare the efficacy and safety of *G. kola 0.5*% aqueous eye drops to that of timolol maleate 0.5% in newly diagnosed patients with primary open-angle glaucoma (POAG) or ocular hypertension (OH). The primary outcome measure was the change in IOP over 24 weeks.

## MATERIALS AND METHODS

### Setting

Patients of either gender recruited from the eye clinics of three tertiary health facilities that fulfilled study criteria comprised the study population.

### Study design and randomization procedure

This was a randomized, double-masked, multicenter, active-controlled clinical trial in patients with primary open angle glaucoma and ocular hypertension. All newly diagnosed adult subjects with bilateral open-angle glaucoma or ocular hypertension were eligible to participate. The research dose assessor, clinical assessors, subjects and ophthalmic surgeons were masked to the allocation of medication and treatment groups. This study complied with the tenets of the Declaration of Helsinki and was conducted in accordance with the ethical standards of Institutional Ethical Review Committee at each investigational centre. Written informed consent was obtained from all study participants. The Pharmacy Council of Nigeria approved and registered *G. Kola* 0.5% aqueous eye drops for human research and consumption.

To diagnose POAG/OH, a detailed history was obtained and clinical examination was performed. Randomization occurred in blocks of two using computer generated random numbers (Excel 5.0, Microsoft Corp., Redmond, Wa., USA). The subjects were randomly assigned (1:1) to receive either timolol maleate 0.5% [Group 1] or *Garcinia kola* 0.5% aqueous solution eye drops [Group 2], twice daily (bid), for a period of 24 weeks.

### Inclusion and exclusion criteria

Patients were eligible if they were at least 18 years old and diagnosed with either POAG or OH based on an average IOP of greater than 21 mmHg consistently for six weeks without treatment. In the POAG group, there were demonstrable typical glaucomatous visual field defects (nasal step, arcuate, para-central, or Seidel's scotoma) detectable by automated threshold perimetry and glaucomatous optic nerve head cupping in the presence of open normal appearing angles. The OH group had no optic nerve or visual field defects.

Patients were excluded if they had any other ocular disease, any systemic disease, were currently using eye medications, had a contraindication to *Garcinia kola*, beta blockers or to any of the components of the medications. Contraindications to *G*. *kola* in this study refers to systemic hypotension, palpitation and dizziness as previously documented.^12^ Other exclusion criteria included, a previous history of ocular trauma or surgery, ocular infection, advanced cataract or inflammation, previous use of corticosteroids, corneal abnormalities, dry eyes or any condition that may interfere with reliable applanation tonometry.

Only one eye that fulfilled all inclusion criteria was designated as the study eye. In cases where both eyes fulfilled all inclusion criteria, the eye designated the study eye was the one with higher IOP. Both eyes were treated with the same medication in patients with simultaneous bilateral involvement. The clinicians and researchers reconciled the subject outcome data. In cases where data and results could not be reconciled, the patient was excluded from data analysis.

### Study masking procedure

At each study center, a consultant ophthalmologist who was not part of the study group performed the initial clinical evaluation of prospective subjects. These ophthalmologists performed the initial diagnoses and were the outcome evaluators of study subjects.

An experienced ophthalmic nurse who was not part of the study group distributed the medication to the study subjects. The nurse was masked to medications in the study and the allocation of individuals to the different groups. At each center, a senior registrar in ophthalmology who was not part of the research group measured IOP, visual acuity, central visual field and performed clinical evaluations. All measurements were standardized across study centers as described below.

Control and research medications were instilled at 6 am and 6 pm daily. Clinicians and subjects were masked to the type of topical medication being instilled into the eye. Clinicians and subjects were masked to the block size until after completion of the study. Subjects were treated for six months with regular follow-up at 2 weeks, 6, 12 and 24 weeks.

### Study subject evaluation

To ensure adequate compliance and adherence to study protocol, each subject was given a drug diary for marking the dosage of each medication. The diary and the marked drug containers were inspected at every follow-up visit.

Goldman applanation tonometry was performed 9 am, 12 pm and 3 pm at the baseline visit, week 6, week 12 and week 24 visits. All subjects underwent visual acuity, gonioscopy and funduscopy (direct and indirect) at each visit. The central visual field was measured in each subject using the AP-125 Kowa full threshold 30-2 visual field analyzer (Kowa Co. Ltd., Tokyo, Japan) at all centers. Each patient had two baseline visual fields performed within 1 month. The central visual field was repeated at 3/12 and 6/12 and subsequently at one year intervals.

During follow-up visits, some of the participants voluntarily commented about possible side effects of the topical medication they were allocated. The clinical evaluators also verbally enquired about possible side effects. All volunteered and elicited reports of adverse events were documented.

### Safety

The subjects who received at least one dose of the study medications were assessed for their safety. Tolerability evaluations consisted of clinical laboratory testing such as hepatic (aminotransferase activities) and renal (serum creatinine) functions, adverse events, and physical examinations. Adverse events reported by the patient or observed by the investigator during clinical evaluation were documented. In addition, patients were questioned at each visit regarding the occurrence of adverse events using a nonspecific question. Safety was assessed by measurement of visual acuity, slit-lamp biomicroscopy, and adverse event reports. Patients could choose to withdraw from the study at any time. If patients were lost to follow-up during the study, data were included for analysis up to the last visit date. Investigators rated the intensity of adverse events and their subjective assessment of the relationship to study medications while masked to the treatment group. Adverse events were significant if they were severe enough to lead to subject withdrawal from the study.

### Statistical analyses

The target sample size of 176 participants (88 participants per group) was determined by assuming that a standard deviation of 3.2 mmHg would provide 90% power and a difference of 1.5 mmHg or 80% power such that a difference of 1.25 mmHg could be excluded between groups.

All analyses were performed with the intention to treat a cohort, defined as all patients who took at least one dose of a study medication. Data were analyzed by using Statistic Package for Social Sciences (SPSS) version 15.0 for windows (SPSS Inc., Chicago, Ill, USA). The comparability of patients in the two groups was determined from the demographic data and baseline IOP. A paired *t*-test was used for parametric comparison of the means. The change in the mean IOP of the groups was evaluated using a 2-way analysis of variance (ANOVA) with post hoc comparison test. The confidence interval (CI) was 95% and *P* < 0.05 was considered statistically significant.

## RESULTS

**Figure 1:**
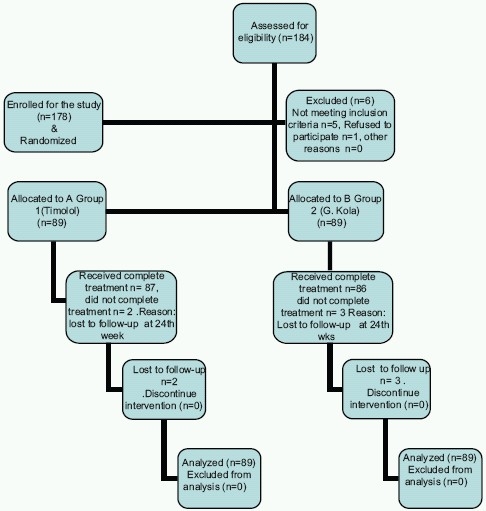
Flow chart showing the total numbers of eyes of patients enrolled, treated and followed for the duration of the study. Data from patients who failed to show for the last follow-up visit were included in the final analysis

One hundred and eighty-four subjects were enrolled in this study with only 178 subjects completing the study. The outcomes of 178 study subjects were analyzed. Six subjects who did not comply with drug administration and follow up were excluded. One hundred and fifty-seven subjects were diagnosed with POAG and 21 with OH. Randomization generated 89 subjects who received *G*. *kola* and 89 subjects who received timolol. Mean age in Group 1 was 48.7 ± 14.6 years and 44.35 ± 12.7 years in Group 2. Age ranged from 18 to 75 years for the study cohort. Forty-one subjects (46.1%) in Group 1 and 40 (44.9%) subjects in Group 2 were below 50 years of age [Figure 1].

Table 1 presents the demographic characteristics of the study subjects. At baseline, gender (*P* = 0.25), age (*P* = 0.32), and VA (*P* = 0.08) did not significantly differ between Group 1 and Group 2.

**Table 1:** Demographics of subjects in Group 1 and Group 2 who received one of two topical medications

Characteristics	Group 1 (Timolol) frequency	Group 2 (*Garcinia kola*) frequency
Age [mean(SD)]	48.7 (SD ± 14.59)	44.35 (SD ± 12.67)
Male	56	52
Female	33	37
Mean blood pressure (mm Hg)		
Systolic	115 (SD ± 10.8)	113 (SD ± 12.3)
Diastolic	91 (SD ± 9.2)	89(SD ± 10.4)

### Intraocular pressure

Mean IOP levels in the two treatment groups were similar at baseline. Mean IOP was 27.17 ± 9.19 mmHg [95% CI, 25.40 mm Hg-28.50 mmHg] in the Group 1 and 27.04 ± 9.22 mmHg [95% CI 25.60 mmHg-28.70 mmHg] in Group 2, [*P* = 0.13, Table 2].

**Table 2:** Comparison of mean intraocular pressure over time in two groups of subjects who received one of two topical medications

Visit period	Group 1 mean IOP (mm Hg)	Standard deviation	Group 2 mean IOP (mmHg)	Standard deviation	Number of patients
Presentation	27.17	9.194	27.04	9.218	72
6 weeks	20.43	6.261	20.57	6.355	72
12 weeks	17.38	6.143	17.73	6.318	69
24 weeks	14.08	8.006	14.11	7.659	65

Group 1 instilled 0.5% topical timolol maleate; Group 2 instilled 0.5% topical *Garcinia kola*

Both treatments significantly reduced the mean diurnal IOP from baseline [*P* = 0.00, Group 1 and *P* = 0.00, Group 2] [Table 3]. The mean IOP reduction at the end of the study period (24^th^ week) was 12.9 ± 2.3 mmHg (47.8% reduction), in Group 1 and 13.1 ± 2.8 mmHg (48.2% reduction) in Group 2. There was no statistically significant difference in IOP reduction between groups [*P* = 0.23, Table 3].

**Table 3:** Comparison of the difference between mean intraocular pressure at 24 weeks and at 6 weeks and 12 weeks in two groups of subjects who received one of two topical medications

Mean IOP	Weeks	Mean difference (mmHg)	Standard error	*P* value	95% CI	
					
					Lower bound	Upper bound
Group 1 (Timolol)						
IOP (n = 89)	24	−12.93	.795	.000	11.37	14.49
	6	−6.47	.795	.000	−8.03	−4.91
	12	−2.84	.795	.000	−4.40	−1.28
						
Group 2 (*G. Kola*)						
IOP (n = 89) subjects	24	−13.09	.795	.000	−14.49	−11.37
	6	−6.46	.795	.000	−8.02	−4.89
	12	−3.62	.795	.000	−5.18	−2.06

Based on observed difference, level of significance was *P* <0.05; group 1 instilled 0.5% topical timolol maleate; group 2 instilled 0.5% topical *Garcinia kola*

**Figure 2:**
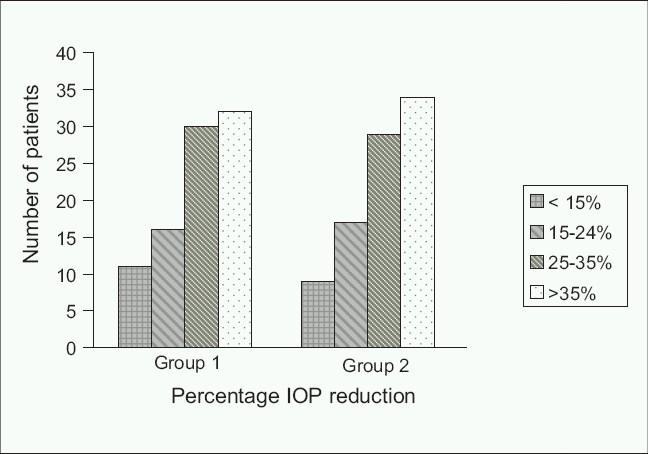
Number of subjects with a decrease in intraocular pressure stratified according to percentage levels of intraocular pressure decrease; Group 1: instilled 0.5% topical timolol maleate; Group 2 instilled 0.5% topical *Garcinia kola;* No significant difference was noted between groups (*P* = 0.23, paired *t* test)

At the end of the study, 39 subjects (43.8%) achieved an IOP lower than 18 mmHg in the Group 1 compared to 41 subjects (46.1%) in Group 2. The number of eyes experiencing stratified according to IOP reduction in both groups is plotted in Figure 2.

There was no significant difference in the number of eyes experience different levels of IOP reduction between the two study groups [*P = *0.23, Figure 2].

### Adverse events

Adverse events in Group 1 were stinging and burning sensation of the eyes (3.4%), conjunctival hyperemia (2.3%) and headaches (2.3%). Less frequently dry eyes, tearing, sticky eyes, and ocular itching was reported in 1.1% if subjects. The most common adverse events in Group 2 were hyperemia (6.7%), sticky eyes (5.6%), stinging and burning sensation of the eyes (4.5%), dry eyes(3.3%), eye discharge (3.4%%) and anorexia (2.3%). Adverse events related to therapy were mild in nature, and there were not statistically significant differences in severity between groups (*P* > 0.05).

## DISCUSSION

The results of the current study indicate that *Garcinia kola* 0.5% aqueous eye drops are as effective as timolol in reducing IOP in newly diagnosed patients with POAG or OH. Additionally the outcomes confirm the efficacy of timolol among Nigerians with POAG and OH. Timolol and *G*. *kola* aqueous eye drops were effective in the reduction of IOP at 6, 12 and 24 weeks. *G*. *kola* had a significant IOP lowering effect at 12 weeks compared to timolol. Chromatography has established that *G*. *kola* extracts have similar features to some commonly used glaucoma medications.^16^,^17^

In the *G*. *kola* group, IOP reduction from baseline to the 6^th^ week was approximately 24% and 25% in the timolol-treated eyes. The mean IOP reduction at 6 months in the *G*. *Kola* group and timolol-treated eyes was 47.8% and 48.2%, respectively with no statistically significant difference between groups (*P >*0.05). The significantly greater IOP lowering effect after six weeks of treatment was due to poor drug compliance initially in the majority of subjects. Most subjects exhausted their assigned drug earlier than expected and stopped further treatment a few days prior to the upcoming follow up. Hence the IOP reduction was minimal six weeks into the study compared to subsequent visits. Furthermore, subjects did not contact the study coordinator for additional medication because they felt that the treatment had no effect. However, were counseled on the importance of continuing medication regardless of their perceptions for the duration of the study and there was better compliance from 6 weeks onwards.

The efficacy reported in the current study is better than the previous reports which examine different IOP lowering medications. For example, randomized, double-masked clinical trials in patients with POAG or OH reported mean IOP reduction of 31% in latanoprost-treated group and 26% in timolol-treated patients over six months.^19^,^20^ This is somewhat lower than the 47.8% and 48.2% reduction seen in Groups 1 and 2 respectively. One possible explanation for this difference is that the subjects in the current study were newly diagnosed and had not undergone medical therapy for glaucoma previously. Therefore, drug resistance, adaptation and possible tolerance to the control and *G*. *kola* eye drops were not encountered compared to patients with a history prolonged use of topical medications.

The IOP lowering effect *G*.*kola* 0.5% aqueous eye drops over a two-week period has been previously demonstrated in animal studies.^13^ A study of guinea pigs, rabbits, sheep and dogs reported a decrease in IOP of normal eyes ranging from 62.9% to 76.2%.^13^

The use of ocular hypotensive medications has been associated with various adverse events in routine practice.^21^–^23^ Systemic side effects such as headaches, cough and allergy can occur in some patients.^24^–^29^ We monitored the safety of treatment through diverse sources of information. Safety data from the current study indicate, there was no evidence of excessive risk in the *G*. *Kola*-treated group. Ocular and systemic effects observed reflected basic physiological responses of various receptors to *G*. *Kola*.^17^ The conjunctival hyperemia and other side effects were likely due to the abnormal sensitivity to kolaviron, a natural biflavonoid in *G*. *kola*.^12^ *G*. *Kola* has antiviral properties, thus subjects with recent viral infections whose symptoms had largely resolved immediately preceding the study could mount a higher immunological reaction compared to others who had no previous exposure to any form of viral infections immediately prior to study inception.

The number of patients with complaints of stinging and burning sensation of the eyes was similar in the two groups. Lid edema related to a high diffusion coefficient gradient for *G*. *kola*, which varies among patients, and is not predictable. The tight junctions of epithelial cell linings are weak which can increase diffusion and precipitate an inflammatory reaction as seen in subjects treated with *G*. *kola eye drops*. Further studies are necessary to definitively determine the cause of this effect.

The metal chelating properties and antioxidant activity of *G*. *Kola* has been reported to protect against the oxidation of lipoprotein.^12^ This observation may account for the stickiness of the eyes when the eye was exposed to a dry environment. Increased eye discharge may be due to vasodilatation.^17^ The relaxation of smooth muscles and analgesic/anti-inflammation property associated with *G*. *kola*^12^ could account for absence of headaches in the *G*. *kola* group. Timolol and *G*. *kola* eye drops are foreign to the eye. Hence, the natural defensive mechanisms mounted against these materials were common events, manifested as foreign body sensation in subjects.

## CONCLUSION

Topical *Garcinia kola* 0.5% aqueous eye drops are as effective as timolol maleate 0.5% eye drops in lowering IOP in newly diagnosed glaucoma and ocular hypertensive patients. The mean IOP reducing efficacy after six months of use was similar in both groups. *Garcinia kola* extract may represent an alternate topical medication for patient with open angle glaucoma and ocular hypertensives in a resource limited population.
